# Tadpole Doctor: a UK-based citizen science project that identifies novel Perkinsea clades closely related to candidate amphibian pathogens

**DOI:** 10.1093/ismeco/ycag154

**Published:** 2026-06-05

**Authors:** Camille Poirier, David S Milner, Estelle S Kilias, Guy Leonard, Varsha Mathur, Emily Brannigan, Miloslav Jirků, Vanessa Smilansky, Frances Orton, Julius Lukeš, Aurélie Chambouvet, Thomas A Richards

**Affiliations:** Department of Biology, University of Oxford, Oxford OX1 3SZ, United Kingdom; Sorbonne Université, CNRS, UMR7144, Station Biologique de Roscoff, 29680 Roscoff, France; Department of Biology, University of Oxford, Oxford OX1 3SZ, United Kingdom; Department of Biology, University of Oxford, Oxford OX1 3SZ, United Kingdom; Department of Biology, University of Oxford, Oxford OX1 3SZ, United Kingdom; Department of Biology, University of Oxford, Oxford OX1 3SZ, United Kingdom; Department of Biology, University of Oxford, Oxford OX1 3SZ, United Kingdom; Institute of Parasitology, Biology Centre, Czech Academy of Sciences, 37005 České Budějovice, Czech Republic; SecureBio, Inc, Cambridge, MA 02142, United States; Institute of Life and Earth Sciences, School of Energy, Geoscience, Infrastructure and Society, Heriot-Watt University, Edinburgh EH14 4AS, United Kingdom; Institute of Parasitology, Biology Centre, Czech Academy of Sciences, 37005 České Budějovice, Czech Republic; Faculty of Sciences, University of South Bohemia, 37005 České Budějovice, Czech Republic; Sorbonne Université, CNRS, UMR7144, Station Biologique de Roscoff, 29680 Roscoff, France; Department of Biology, University of Oxford, Oxford OX1 3SZ, United Kingdom

**Keywords:** amphibian conservation, protist diversity, long-read rDNA sequencing, pathogen emergence, severe Perkinsea infections

## Abstract

Parasites of the Perkinsea lineage infect both unicellular eukaryotic and metazoan hosts. The Pathogenic Perkinsea Clade (PPC) has been associated with Severe Perkinsea Infection (SPI) and mortality events in wild tadpole populations across the USA and in captive European Tree Frogs (*Hyla arborea*) in the UK. To investigate the spread of this emergent disease and identify additional putative parasites, we launched a UK-based citizen science project called ‘Tadpole Doctor’. Volunteer participants collected tadpoles, sediment samples from ponds suspected to contain tadpoles, or alternatively, sediment from ponds where there was evidence of recent loss of a tadpole population. In addition to the UK samples, tadpoles with SPI symptoms were collected in Alaska and Florida, along with asymptomatic tadpoles in the Czech Republic. To identify traces of PPC and related lineages, we developed a semi-targeted long-read small-subunit (SSU) — Internal Transcribed Spacer (ITS) rDNA sequencing approach, revealing an unprecedented molecular diversity of the wider Perkinsea clade NAG01, where PPC branches in SSU rDNA phylogenies. Seven groups of sequences other than PPC were found associated to tadpole livers, potentially reflecting novel parasitic infections. We also identified three sister groups to the PPC clade, among which one group was found in a tadpole, and another was frequently encountered in environmental DNA metabarcoding surveys. Deeper characterization of this diversity may identify new threats to amphibians. Overall, these data illustrate how citizen-based research can be useful for assessing organismal distribution patterns, enabling further targeted studies, while also raising public awareness of the vulnerability of amphibians to infectious disease.

All described Perkinsea (Alveolata) species are putative parasites and infect a broad range of organisms including unicellular hosts (microalgae) and metazoans (molluscs, fish, and amphibians) [1]. These protists form diverse and globally distributed lineages [2], yet many of the identified Perkinsea clades are entirely composed of eDNA sequences [2–5]. The ecology of these Perkinsea groups remains obscure. However, Severe Perkinsea Infection (SPI) has been associated with members of the Pathogenic Perkinsea Clade (PPC) and linked to mass mortality events in tadpole populations across the USA [6]. SPI is estimated to represent one of the three most commonly sampled infections of amphibians [6, 7]. This Perkinsea–host association was first identified in 1999 in New Hampshire [8] and has now been reported in numerous USA states, from Alaska to Florida [6, 9]. PPC sequences were also obtained from liver-tissues of tadpoles from Panama and captive *Hyla arborea* specimens in the UK, expanding our understanding of the geographic and host association of this group [10].

To study the spread of PPC and other putative Perkinsea parasites associated with tadpoles in the UK, we developed a new approach for eDNA sequence sampling for the Perkinsea and launched a citizen-science project called ‘Tadpole Doctor’ (https://tadpole-doctor.co.uk/, see logo—Fig. 1) to sample a wide range of relevant habitats. Volunteer participants collected sediment from 25 aquatic habitats containing suspected non-symptomatic tadpole populations, or from 5 aquatic habitats which had recently lost evidence of an active tadpole population (Fig. S1, Table S1). In addition, volunteers collected 32 tadpoles across 8 sites in England that were found dead and/or showing advanced SPI-like symptoms (i.e. bloating around abdomen, abnormal swimming, skin discolouration [11]), and 13 non-symptomatic tadpoles originating from three sites in Scotland (Fig. S1, Table S2). Samples were collected according to safety guidelines provided in the sampling kits and were preserved in LifeGuard™ Solution (Qiagen) at site, without disturbing the natural environment (Supplementary Methods). Other samples collected in the UK prior to the Tadpole Doctor project were added to the study and included sediment from 4 ponds in the Lake District area of the UK (Table S1), as well as archived tissue samples from captive *H. arborea* collected in 2019 (Table S2), where PPC was detected [10]. Tadpoles originating from the same sampling event were pooled for downstream processing.

**Figure 1:**
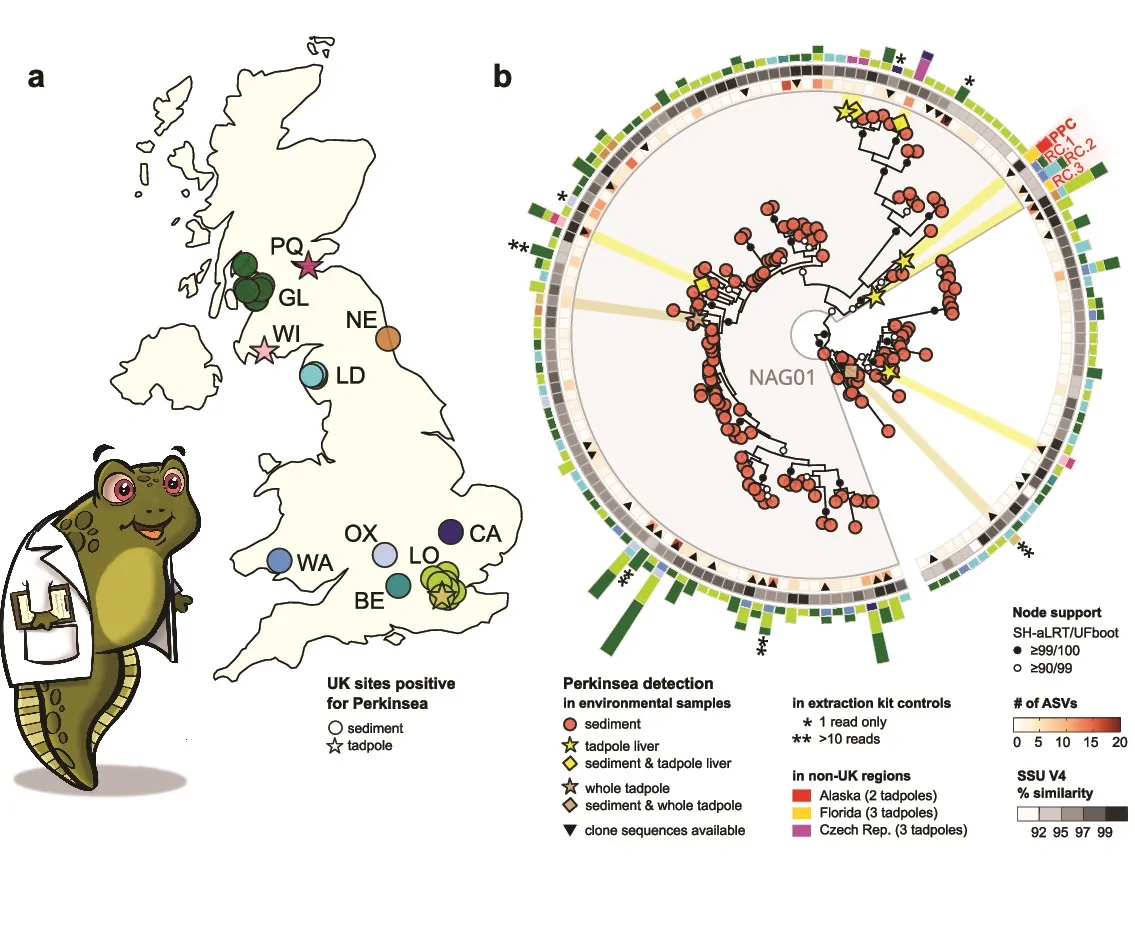
Sampling locations positive for Perkinsea in the UK and a phylogeny of the Perkinsea sequences detected in this study. (a) Logo for Tadpole Doctor project is shown next to the UK sampling map. The map illustrates the geographic range of participants and samples positive for Perkinsea in the UK (see Fig. S2 for total sites sampled, including those negative for Perkinsea). Sediment samples are represented by circles (see also Table S1), and tadpole samples are represented by stars (see also Table S2). Different colours indicate regional sampling locations (PQ = Prestonhill Quarry, GL = around Glasgow, WI = Wigtown, NE = Newcastle, LD = Lake District, WA = Wales, OX = Oxfordshire, CA = Cambridge, LO = around London, BE = Berkshire). (b) Maximum-likelihood phylogeny of Perkinsea derived from a concatenated alignment of the SSU and 5.8S rRNA gene regions detected in this study (150 sequences, 1438 positions, GTR + F + I + G4 model, SHaLRT/UFboot support with 1000 replicates). Each branch represents an ITS sequence type (clustered at 97% similarity threshold; see Supplementary methods). One ITS type was excluded from the phylogeny because it represented a long-branch sequence. Each ITS type is coloured according to its biological provenance (dark orange dots for sediment samples, yellow stars for tadpole livers, yellow diamonds when found in both sediment and tadpole livers, beige stars for whole tadpoles, beige diamonds when found in both sediment and whole tadpoles). The tree is rooted based on the two most basal sequences in a reference SSU phylogeny (see Fig. S3) and the NAG01 clade is highlighted with a grey background. The number of ASVs belonging to each ITS sequence type is shown in the orange gradient (inner ring) and the presence of clone sequences is indicated with black triangles. The SSU V4 rRNA region of each ITS sequence type representative was BLASTn searched against NCBI on 20th March 2026 (results shown in the central ring shaded in grey—see % similarity key in the bottom corner). Number of sampling sites where ITS sequence types are found are represented in the outer histogram (ranging from 1 to 13). Sites are coloured according to locations on the UK map in (a), with additional sampling locations for tadpoles in Alaska (red), Florida (orange) and Czech Republic (magenta). Asterisks mark the presence of seven ITS sequence types in kit extraction control samples (see Supplementary methods and Fig. S4). Single asterisks indicate these represent single read sequences in the kit control samples, while double asterisks indicate they represent at least 10 reads in those samples.

We then performed DNA extractions, PCR amplification of a 2 kb region encompassing the near-complete small-subunit (SSU) rRNA encoding gene and the complete Internal Transcribed Spacer (ITS) region [12], clone library construction combined with Sanger sequencing, and, separately, parallel-tag sequencing of the target amplicons using a Sequel II platform (Pacific Biosciences) (Fig. S2). This approach allowed for analysis of freshwater Perkinsea in the UK, focusing particularly on the NAG01 clade, which contains PPC and additional putative tadpole-associated Perkinsea [13] (Fig. S3). Previous studies using the SSU rRNA gene showed little biogeographical structure within PPC [10]. Therefore, we combined the SSU and ITS sampling, as the ITS region tends to show a faster evolutionary rate [14] and can provide finer resolution for biogeographic studies [15, 16]. We also obtained Perkinsea SSU-ITS rRNA gene sequences from single tadpole livers from two specimens collected in Alaska during an SPI outbreak (2016) [9], from three symptomatic specimens collected in Florida (2019), and from three non-symptomatic specimens collected in the Czech Republic (2022) (Table S2).

We clustered 540 Perkinsea Amplicon Sequence Variants (ASVs) at 97% identity (Supplementary methods), identifying 151 ‘ITS types’ (Fig. 1). Half of the Perkinsea sequence types shared less than 97% identity to sequences available in NCBI for the SSU V4 region (BLASTn, March 2026), suggesting the targeted approach is useful for sampling novel, previously upsampled, Perkinsea diversity. Our sequencing did not detect PPC sequences in the UK, although we recovered SSU-ITS sequences of PPC in tadpoles from Alaska and Florida. We also detected three related clades (labelled ‘RC’) that branch close to PPC with strong phylogenetic support: RC.1 and RC.2 from UK sediment (in two and four ponds, respectively) and RC.3 recovered from a tadpole from Florida. Seven other ITS sequence types were found associated with tadpoles, including three ITS types from the Czech tadpole livers, two ITS types that co-occurred in UK tadpole livers, and two ITS types detected in archived whole *H. arborea* tadpoles. All seven additional tadpole-associated ITS types clustered in distant phylogenetic positions to PPC (Fig. 1).

In addition, seven distinct Perkinsea ITS-types were detected in the ‘blank’ DNA extraction kit-controls (see Supplementary Methods). These sequences do not include PPC or RC.1–3, and do not significantly change our understanding of the distribution of Perkinsea observed in the environmental samples (Fig. S4) but highlight the importance of controls in eDNA processing for revealing background Perkinsea contamination in commercial DNA processing kits [17].

Recovery of PPC-related clades in Eukbank [18] and Eukaryome [19] environmental databases suggest many of the groups detected are globally distributed (Fig. 2a). Specifically, RC.2 was the most frequently detected clade, with sequences generally recovered in colder environments/latitudes (i.e. a glacial lake in Spain, boreal forests of Alberta and Norway, the Lake District in the UK), while RC.1 was detected in Scotland, Wales, and Costa Rica. While their potential for infection and disease association cannot be inferred here, it is interesting to observe that RC.1 and RC.2 were detected in a single pond where tadpole mortality was recorded by a volunteer participant in our study (Fig. 2b). Two Eukaryome environmental sequences from a permafrost pond in Canada and from soil in northern Sweden form distinct branches in the SSU-ITS phylogeny, indicating an extended diversity of the ‘RC’ group (Fig. 2b).

**Figure 2:**
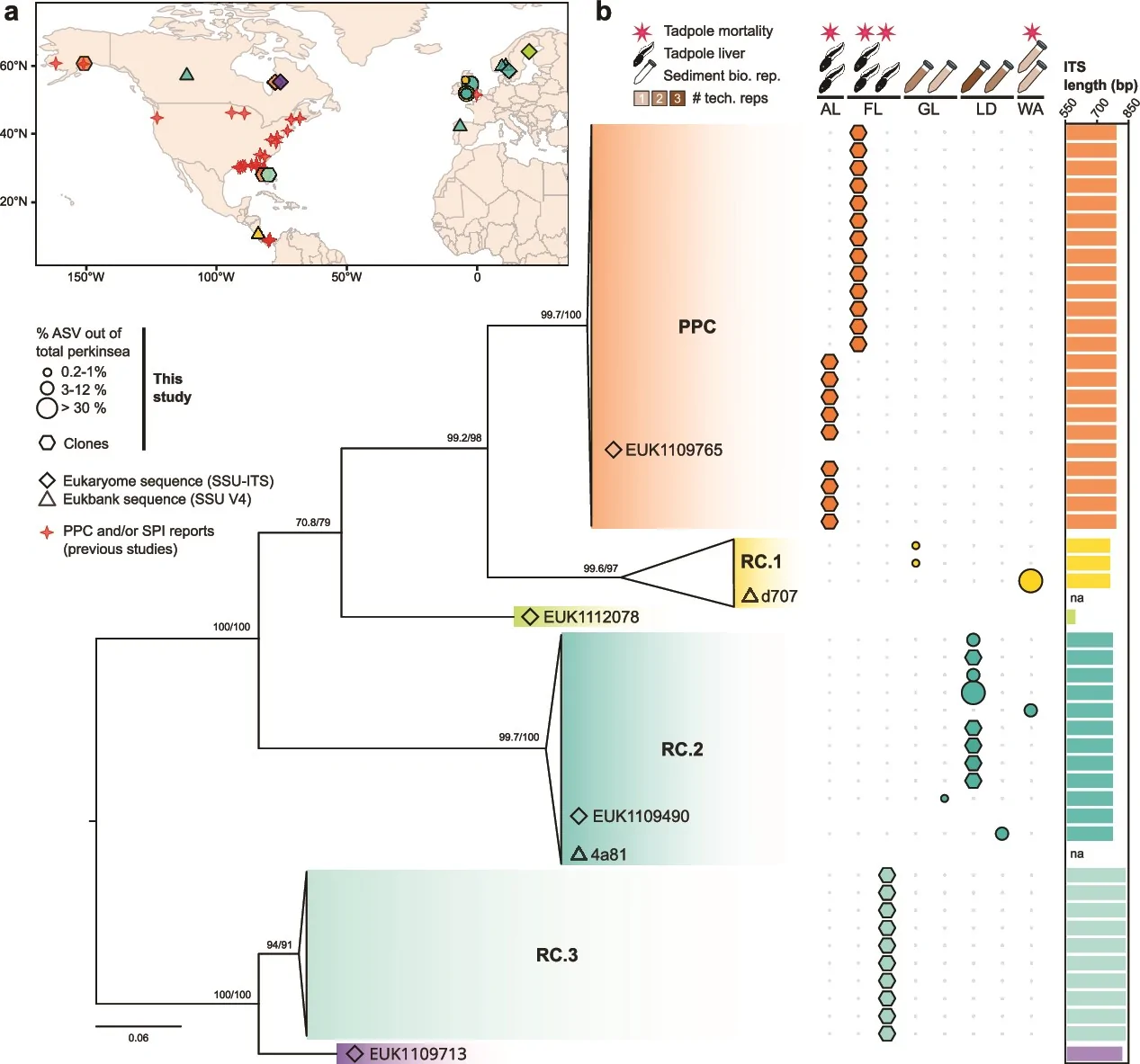
Geographical distribution and phylogeny of PPC and related clades. (a) Map showing the distribution of clone sequences (hexagons) and ASVs (circles) obtained in this study. Circle size is relative to the contribution of each ASV to total Perkinsea ASVs at each sampling site. Environmental SSU-ITS sequences retrieved from the Eukaryome database are shown with diamonds, and partial SSU sequences from the Eukbank database (V4 region) with triangles. Colours of the symbols refer to the clades/branches on the phylogeny in (b). Four pointed red stars indicate previous reports of PPC or SPI. Two Eukaryome sequences from Canada and two clone sequences from Florida are slightly offset for visualization but originate from the same site. (b) Maximum likelihood phylogeny of partial SSU + ITS of PPC and the related clades (2169 positions, TVM + F + G4 model, SHaLRT/UFboot support with 1000 replicates). Symbols representing the origin of sequences in the phylogeny are illustrated using the same conventions as the map (a). Two Eukaryome sequences originating from Sweden and Canada form distinct branches on the phylogeny. Only the first four digits of the Eukbank sequence IDs are included for simplification. The length of the ITS region (bp) is shown as a histogram and highlights this marker variability between the clades. Tadpoles were sampled in Alaska (AL) and Florida (FL), and sediment in the UK around Glasgow (GL), in the Lake District (LD) and in Wales (WA), as shown above the phylogeny. Number of tadpoles above sequences originating from Alaska and Florida mark the number of independent tadpole livers where clones were found. Number of sediment tubes above sequences originating from the UK mark the number of sediment biological replicates where clones or ASV sequences were found, while the gradient brown colour inside the tubes shows the number of PCR technical replicates (associated to each biological replicate) where those sequences were found (see experimental workflow in Fig. S2). At each site, the percentage of each ASV out of total Perkinsea ASVs was calculated within each technical replicate, then averaged if found in more than one technical replicate. For the site in Wales, one biological replicate contained 1 ASV of RC.1 and a second one contained 1 ASV of RC.2 (both found in single technical replicates). In that case, the percentage of each ASV was calculated independently within each technical replicate and merged on the same column. Pink stars above tadpoles and sediment tubes indicate that tadpole mortality was reported at the time of sampling.

The ITS region differs dramatically between the different clades sampled (PPC, RC.1–3) showing 60% variable sites across the DNA alignment, while being relatively similar in size (except for a Eukaryome sequence which is much shorter). On the contrary, the PPC sequences originating from tadpoles from Alaska and Florida display minimal variability in their ITS sequence (2 bp difference) suggesting the biogeographical dispersal between subtropical and subarctic North America encompassed little variation in this marker gene. Retracing the evolutionary dispersal of this organism at the population level may require sampling DNA markers with a higher rate of variation, or alternatively the biogeographic spread of PPC across the USA has been so rapid that there is little or no recoverable sequence variation. Such a pattern would be consistent with rapid parasite emergence [6].

Our findings expand the known diversity of Perkinsea and identify additional clades closely related to Perkinsea, which are putatively associated with tadpole infections. Our analyses did not recover molecular evidence for PPC in natural environmental samples in the UK. However, our citizen science project enabled us to screen numerous samples across a mixture of UK biogeographies. The absence of PPC detection does not prove that this group is absent in the UK, but may suggest that it is not an endemic group. The UK-based ‘Tadpole Doctor’ citizen-science project uncovered molecular diversity suggesting that RC.1/2 may occur in environments associated with tadpoles (and possibly tadpole loss), although the ecologies of these protist groups remain obscure. While phylogenetically related to PPC, the RC clades may represent free-living protists or parasites/symbionts of other hosts. Confirming tadpole association for PPC and RC.1/2 will require targeted ecological and experimental culture-based infection studies. Future work must include broader surveys combining additional sampling in the USA and Europe, with under-sampled regions such as natural habitats in the Southern hemisphere. Future work must also make use of multi-marker gene sequencing, which is needed to understand the spread of these groups in the context of amphibian decline. This study demonstrates that citizen-science projects can facilitate larger-scale biodiversity assessments, support method development, and lay the groundwork for future targeted environmental studies.

## Supplementary Material

Supplementary_material_ycag154

## Data Availability

Demultiplexed fastq files from each run of PacBio sequencing (oriented reads with primers trimmed) are available at the EMBL-EBI repository under ENA project PRJEB103017 (https://www.ebi.ac.uk/ena). Clone sequences are deposited under the same ENA project with accessions OZ370521-OZ370657. These accessions include five additional clone sequences that were not used for generating the phylogenies in this study (they were obtained from a sample consisting of pooled livers from 9 Alaska tadpoles—sample KNADNA—and were identical to clone sequences obtained from single Alaska tadpoles). The sequence alignments used to calculate the phylogenies are available at Figshare (doi:10.6084/m9.figshare.30800921).
