# Supplementary material for: Tadpole Doctor: a UK-based citizen science project that identifies novel Perkinsea clades closely related to candidate amphibian pathogens
Source: ISME Commun. 2026 Jun 5;6(1):ycag154. doi: 10.1093/ismeco/ycag154 (PMC13584925; doi:10.1093/ismeco/ycag154)
Supplement: Supplementary file 1 [file supplementary_material_ycag154.zip › FigS1_28Apr26_ycag154.pdf]

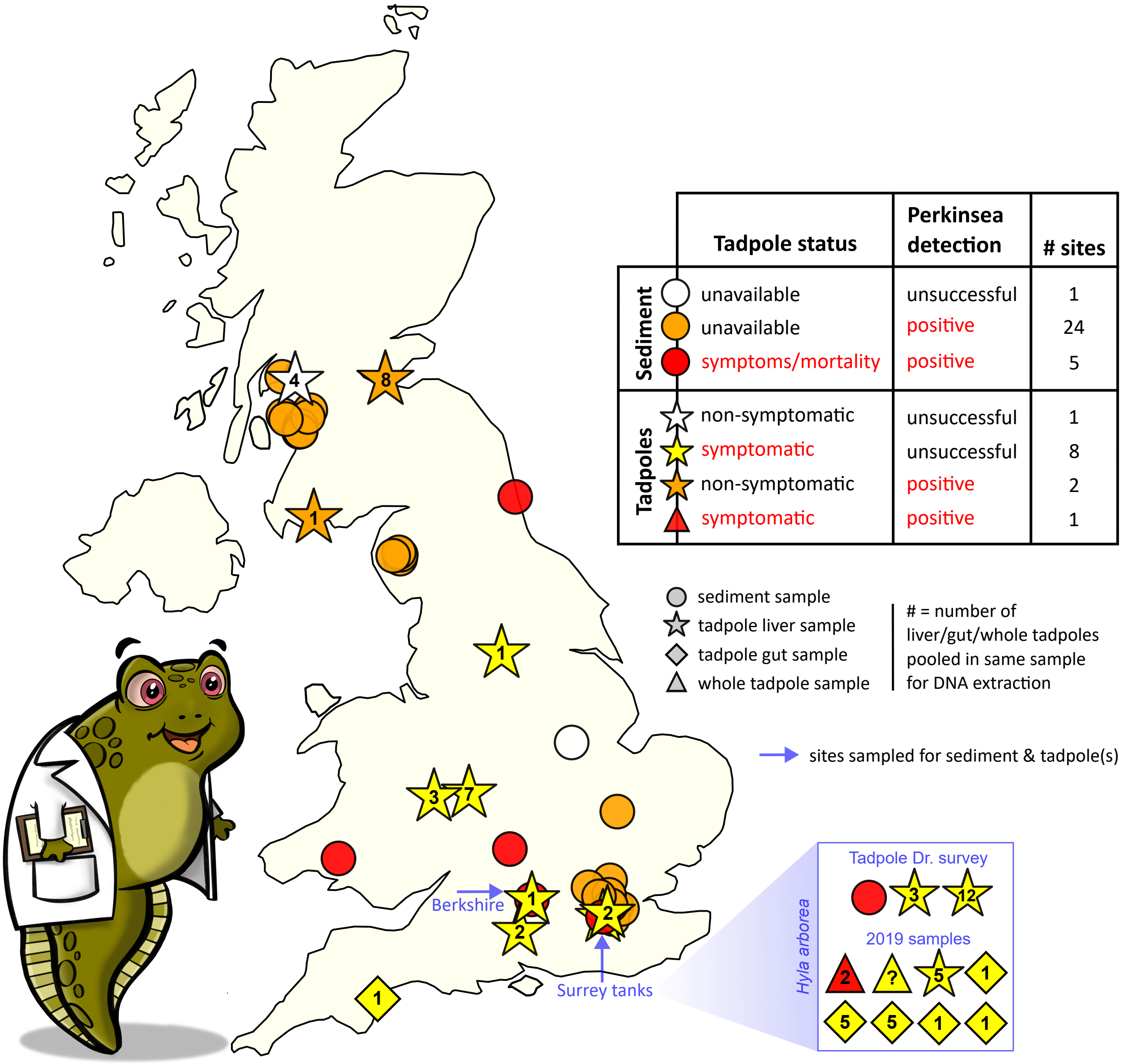

Tadpole status
Perkinsea detection
sites
Sediment
unavailable
unsuccessful
1
unavailable
positive
24
symptoms/mortality
positive
5
Tadpoles
non-symptomatic
unsuccessful
1
symptomatic
unsuccessful
8
non-symptomatic
positive
2
symptomatic
positive
1
sediment sample
tadpole liver sample
tadpole gut sample
whole tadpole sample
# = number of liver/gut/whole tadpoles pooled in same sample for DNA extraction
sites sampled for sediment & tadpole(s)
Berkshire
Surrey tanks
Tadpole Dr. survey
2019 samples
Hyla arborea
