# Supplementary material for: Tadpole Doctor: a UK-based citizen science project that identifies novel Perkinsea clades closely related to candidate amphibian pathogens
Source: ISME Commun. 2026 Jun 5;6(1):ycag154. doi: 10.1093/ismeco/ycag154 (PMC13584925; doi:10.1093/ismeco/ycag154)
Supplement: Supplementary file 1 [file supplementary_material_ycag154.zip › FigS2_28Apr26_ycag154.pdf]

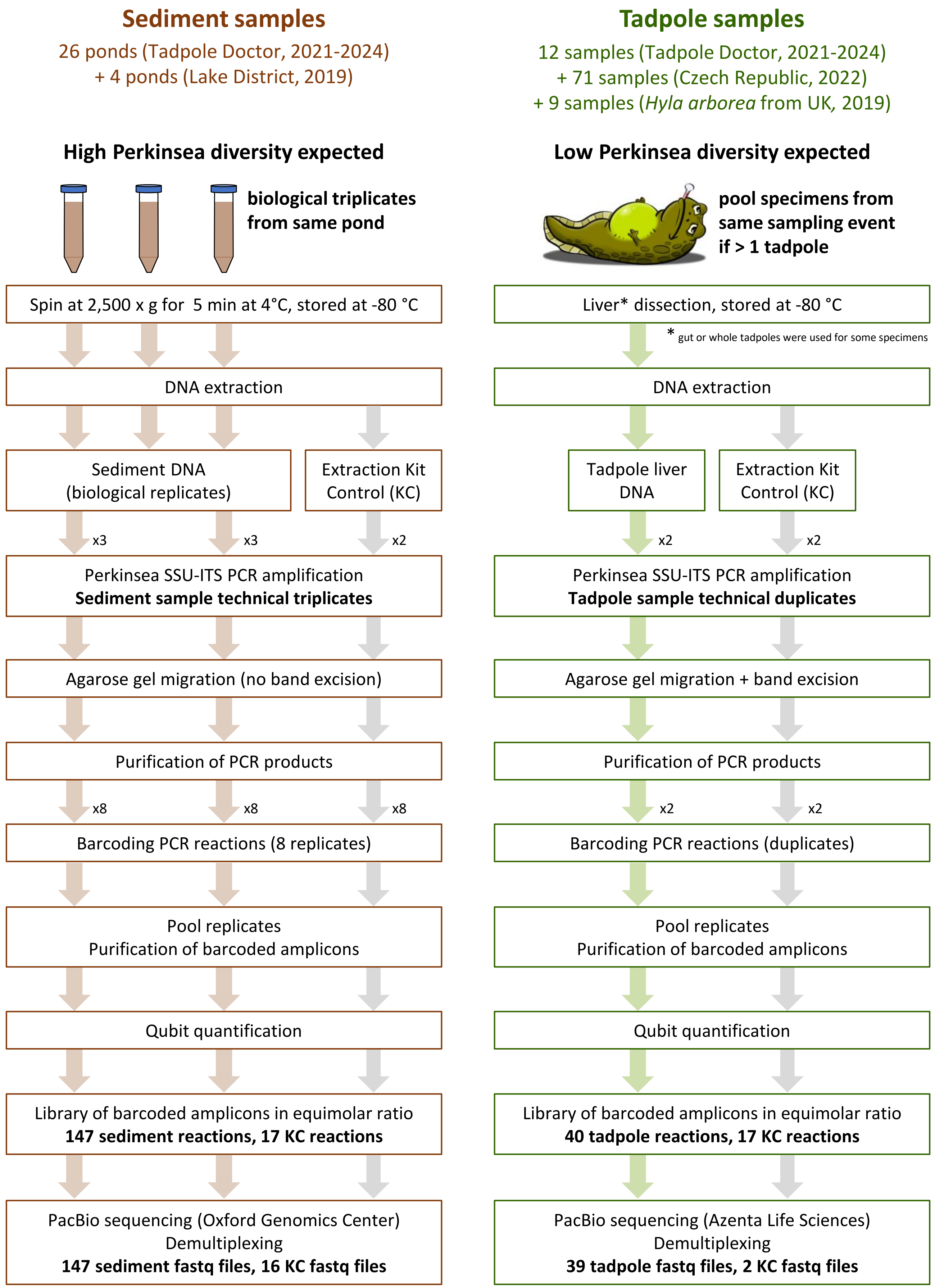
Sediment samples
26 ponds (Tadpole Doctor, 2021-2024)
+ 4 ponds (Lake District, 2019)
High Perkinsea diversity expected
biological triplicates from same pond
Spin at 2,500 x g for 5 min at 4°C, stored at -80 °C
DNA extraction
Sediment DNA (biological replicates)
Extraction Kit Control (KC)
x3
x3
x2
Perkinsea SSU-ITS PCR amplification
Sediment sample technical triplicates
Agarose gel migration (no band excision)
Purification of PCR products
x8
x8
x8
Barcoding PCR reactions (8 replicates)
Pool replicates
Purification of barcoded amplicons
Qubit quantification
Library of barcoded amplicons in equimolar ratio
147 sediment reactions, 17 KC reactions
PacBio sequencing (Oxford Genomics Center)
Demultiplexing
147 sediment fastq files, 16 KC fastq files
Tadpole samples
12 samples (Tadpole Doctor, 2021-2024)
+ 71 samples (Czech Republic, 2022)
+ 9 samples (Hyla arborea from UK, 2019)
Low Perkinsea diversity expected
pool specimens from same sampling event if > 1 tadpole
Liver* dissection, stored at -80 °C
* gut or whole tadpoles were used for some specimens
DNA extraction
Tadpole liver DNA
Extraction Kit Control (KC)
x2
x2
Perkinsea SSU-ITS PCR amplification
Tadpole sample technical duplicates
Agarose gel migration + band excision
Purification of PCR products
x2
x2
Barcoding PCR reactions (duplicates)
Pool replicates
Purification of barcoded amplicons
Qubit quantification
Library of barcoded amplicons in equimolar ratio
40 tadpole reactions, 17 KC reactions
PacBio sequencing (Azenta Life Sciences)
Demultiplexing
39 tadpole fastq files, 2 KC fastq files
