# Supplementary material for: Tadpole Doctor: a UK-based citizen science project that identifies novel Perkinsea clades closely related to candidate amphibian pathogens
Source: ISME Commun. 2026 Jun 5;6(1):ycag154. doi: 10.1093/ismeco/ycag154 (PMC13584925; doi:10.1093/ismeco/ycag154)
Supplement: Supplementary file 1 [file supplementary_material_ycag154.zip › FigS3_28Apr26_ycag154.pdf]

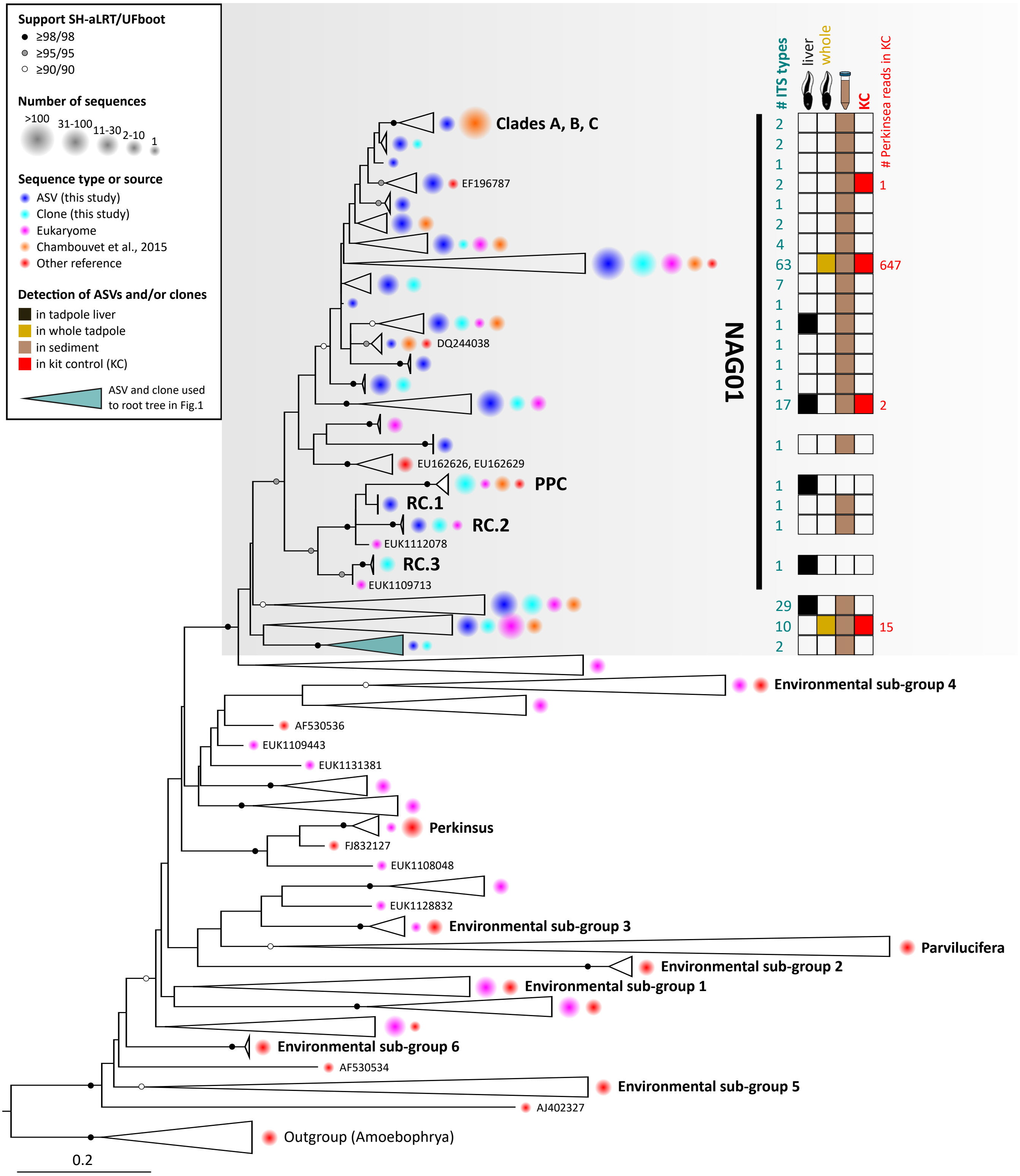
Support SH-aLRT/UFboot
≥98/98
≥95/95
≥90/90
Number of sequences
>100
31-100
11-30
2-10
1
Sequence type or source
ASV (this study)
Clone (this study)
Eukaryome
Chambouvet et al., 2015
Other reference
Detection of ASVs and/or clones
in tadpole liver
in whole tadpole
in sediment
in kit control (KC)
ASV and clone used to root tree in Fig.1
ITS types
liver
whole
KC
Perkinsea reads in KC
Clades A, B, C
EF196787
DQ244038
EU162626, EU162629
PPC
RC.1
RC.2
EUK1112078
RC.3
EUK1109713
NAG01
2
2
1
2
1
1
2
4
63
647
7
1
1
1
1
1
17
2
1
1
1
1
1
29
10
15
2
Environmental sub-group 4
AF530536
EUK1109443
EUK1131381
Perkinsus
FJ832127
EUK1108048
EUK1128832
Environmental sub-group 3
Parvilucifera
Environmental sub-group 2
Environmental sub-group 1
Environmental sub-group 6
AF530534
Environmental sub-group 5
AJ402327
Outgroup (Amoebophrya)
0.2
