# Supplementary material for: Tadpole Doctor: a UK-based citizen science project that identifies novel Perkinsea clades closely related to candidate amphibian pathogens
Source: ISME Commun. 2026 Jun 5;6(1):ycag154. doi: 10.1093/ismeco/ycag154 (PMC13584925; doi:10.1093/ismeco/ycag154)
Supplement: Supplementary file 1 [file supplementary_material_ycag154.zip › FigS4_28Apr26_ycag154.pdf]

AT PERKINSEA ASV LEVEL

Sample id	Location	Date of DNA extraction	Date of band excision from agarose gel	SAMPLE TYPE	PerkASV_001	PerkASV_019	PerkASV_038	PerkASV_101	PerkASV_164	PerkASV_200	PerkASV_327	PerkASV_354	PerkASV_565	PerkASV_575	PerkASV_576
KC-260722	-	26 July 2022	-		0	0	0	0	0	0	0	0	0	1	0
W019	Glasgow area	29 July 2022	-		0	0	135	0	0	0	0	0	0	0	0
KC-160822	-	16 Aug 2022	-		0	0	0	0	0	0	0	0	0	0	1
KC-071022	-	07 Oct 2022	22 July 2024		15	0	0	0	0	0	0	0	0	0	0
KC-060223	-	06 Feb 2023	-		0	1	0	0	0	0	0	0	0	0	0
W074	Cambridge	06 Feb 2023	-		0	4238	0	0	0	0	0	0	0	0	0
W007	Glasgow area	06 Feb 2023	-		0	0	774	173	0	0	0	0	0	0	0
W008	Glasgow area	06 Feb 2023	-		0	0	525	0	0	0	0	0	0	0	0
KC-020523	-	02 May 2023	-		0	0	70	177	182	122	49	44	3	0	0
DM-LD1-2	Lake District	02 May 2023	-		152	0	0	0	0	0	0	0	0	0	0
HA12-new	Surrey tanks	15 July 2024	16 Aug 2024		13	0	0	0	0	0	0	0	0	0	0

AT PERKINSEA ITS-TYPE LEVEL

Sample id	Location	Date of DNA extraction	Date of band excision from agarose gel	SAMPLE TYPE	ITS-type_1	ITS-type_44	ITS-type_68	ITS-type_78	ITS-type_122	ITS-type_134	ITS-type_136
W002	Oxfordshire	18 Jan 2022	-		0	0	0	0	11325	0	0
KC_260722	-	26 July 2022	-		0	0	0	0	0	0	1
W016	Glasgow area	26 July 2022	-		0	0	0	0	0	0	18995
W015	Glasgow area	29 July 2022	-		0	0	0	93	0	0	0
W019	Glasgow area	29 July 2022	-		0	135	0	0	0	0	0
W058	London area	29 July 2022	-		0	0	1693	0	0	0	0
W076-W078	Surrey tanks	12 Aug 2022	-		0	0	4556	0	0	0	0
W069	Glasgow area	15 Aug 2022	-		0	0	0	51	0	0	0
KC_160822	-	16 Aug 2022	-		0	0	0	0	1	0	0
KC_071022	-	07 Oct 2022	22 July 2024		15	0	0	0	0	0	0
KC_060223	-	06 Feb 2023	-		0	0	0	0	0	1	0
W074	Cambridge	06 Feb 2023	-		0	0	0	0	0	7846	0
W007	Glasgow area	06 Feb 2023	-		0	774	4516	0	0	0	278
W008	Glasgow area	06 Feb 2023	-		0	525	0	836	0	0	0
KC_020523	-	02 May 2023	-		0	195	359	93	0	0	0
W081	Wales	02 May 2023	-		0	0	89	0	0	0	0
DM-LD1	Lake District	02 May 2023	-		332	0	0	0	0	0	0
HA12-new	Surrey tanks	15 July 2024	16 Aug 2024		13	0	0	0	0	0	0

- Sediment sample (SMRT-SEDIMENT library)
- Kit control sample (SMRT-SEDIMENT library)
- Tadpole sample (SMRT-TADPOLE library)
- Kit control sample (SMRT-TADPOLE library)

- Perkinsea contamination in KC is not suspected to influence Perkinsea diversity recovered in environmental samples
- Perkinsea contamination in KC is suspected to have a minimal impact on Perkinsea diversity recovered in environmental samples
- Perkinsea contamination in KC is suspected to have an impact on Perkinsea diversity recovered in environmental samples
